# Comparative Study of Mastectomy Surgical Techniques Followed by Reconstruction: Hydrodissection and Electrical Plasma Surgery

**DOI:** 10.3390/jcm14041338

**Published:** 2025-02-18

**Authors:** Silvia Actis, Giulia Lavalle, Stefania Agus, Elena Paradiso, Francesca Accomasso, Carola Minella, Luca Giuseppe Sgro, Mario Boltri, Paolo Balocco, Annamaria Ferrero, Valentina Elisabetta Bounous

**Affiliations:** 1 Gynecology and Obstetrics Unit, Mauriziano Umberto I Hospital, Department of Surgical Sciences, University of Turin, Largo Filippo Turati, 62, 10128 Turin, Italy; silvia.actis@unito.it (S.A.); giulia.lavalle@unito.it (G.L.); stefania.agus@edu.unito.it (S.A.); elena.paradiso@unito.it (E.P.); francesca.accomasso@unito.it (F.A.); carola.minella@unito.it (C.M.); lsgro@mauriziano.it (L.G.S.); annamaria.ferrero@unito.it (A.F.); 2 Plastic Surgery Unit, Mauriziano Umberto I Hospital, 10128 Turin, Italy; mario.boltri@libero.it (M.B.); pbalocco@mauriziano.it (P.B.)

**Keywords:** mastectomy, breast cancer, BRCA

## Abstract

**Background/Objectives**: Mastectomy is a surgical option for breast cancer when conservative treatment is unsuitable, and it is also performed prophylactically in high-risk women. Various surgical techniques can be used for mastectomy, including electrosurgery, which can cause thermal damage to tissues, reducing surgical precision and delaying wound healing. This study aims to compare electrical plasma surgery and hydrodissection, which appear to be the least traumatic methods, to determine the better option for performing mastectomy with immediate reconstruction. **Methods**: Conducted at the “Breast Unit” of AO “OrdineMauriziano Umberto I”, this study analyzed 56 patients undergoing 65 mastectomies (9 bilateral, 47 unilateral). A total of 16 were prophylactic, and 49 were oncologic. All patients received immediate subpectoral reconstruction. Data collected included preoperative medical history, pain, drain flow, blood transfusions, hemoglobin levels, and hospital stay duration. Complications were graded using the Clavien-Dindo classification. **Results**: Both groups were similar in age, body mass index (BMI), smoking habits, and comorbidities. Patients who underwent hydrodissection reported more pain on the first and second postoperative day and had longer hospital stays. The drop in hemoglobin from pre- to postoperative and the volume of surgical drains on the day of surgery and the first and second postoperative days were comparable between groups. Early complications and reintervention rates (Clavien-Dindo grade 3) were similar between techniques. **Conclusions**: Electrical plasma surgery offers better early postoperative outcomes in terms of pain and hospital stay, although overall complication and reintervention rates are unaffected by the technique used. Larger randomized studies are needed to confirm these findings and optimize patient management.

## 1. Introduction

Breast cancer (BC) is the most frequently diagnosed cancer and the leading cause of cancer death among women worldwide [1]. Early-stage BC treatment typically involves either conservative surgery combined with radiotherapy or mastectomy. Mastectomy is indicated when conservative surgery is not suitable, when it is contraindicated (e.g., previous radiotherapy to the wider breast), or when the patient refuses a conservative approach. It is also used prophylactically for women at high risk of developing BC [2]. Currently, the preferred mastectomy techniques are those that preserve the skin (skin-sparing mastectomy, SSM) and, if possible, the nipple–areola complex (nipple-sparing mastectomy, NSM). These techniques are particularly suitable for immediate reconstruction performed during the same surgical session as the mastectomy [3].

The viability of mastectomy flaps is crucial for the success of the reconstruction process. Ischemic flaps increase the risk of complications such as scarring, delayed adjuvant therapy, or breast implant removal, which may lead to increased hospitalization rates and costs [4,5].

Various surgical techniques can be used for mastectomy, including electrosurgery, electrical plasma surgery (EPS), and hydrodissection (HD).

Traditional electrosurgery devices operate at high temperatures (250–350 °C) to achieve effective cutting and hemostasis, but this high heat often causes extensive collateral thermal damage to surrounding tissues [6,7]. In SSM or NSM, this damage can compromise blood flow to the skin flaps, increasing the risk of complications such as partial flap necrosis or delayed wound healing. In addition, thermal injury to the subcutaneous microvascular supply during electrosurgical flap dissection may contribute to partial necrosis of the skin flap and/or the areola–nipple complex, a relatively common complication affecting approximately 10–20% of patients [8]. This complication represents a significant source of postoperative morbidity and an obstacle to nipple preservation and optimal cosmetic outcomes [9].

HD, also known as tumescence dissection, consists of injecting a crystalloid solution into the subcutaneous and pre-pectoral tissues to facilitate dissection and minimize trauma, with the option of adding local anesthetic with epinephrine [10]. The mastectomy is then performed with a cold blade (e.g., scissors) without the use of heat, except where coagulation is required. This technique, pioneered in mastectomy by Worland in 1996, creates a clear dissection plane that allows surgeons to better distinguish between tissue types and reduces intraoperative blood loss [11,12].

EPS is a modern electrosurgical tool that uses low-temperature pulsed radiofrequency energy to cut and coagulate tissues with minimal thermal spread (40–140 °C), reducing collateral tissue damage by 50–90%. Unlike conventional electrocautery, it allows precise cutting with limited inflammation and scarring, as well as reduced wound drainage and seroma formation [7,13].

This study aims to compare EPS and HD, which appear to be the least traumatic dissection methods to improve the reconstructive phase and outcomes, in order to determine the better option for performing mastectomy with immediate reconstruction, focusing on maximizing flap perfusion and minimizing complications.

## 2. Materials and Methods

This retrospective study was performed at the “Breast Unit” of the AO “Ordine Mauriziano Umberto I” of Turin on 56 patients who underwent unilateral or bilateral mastectomy (surgery including the removal of pectoral fascia) with immediate subpectoral plastic reconstruction. All patients routinely signed an informed consent at the time of enrollment for the anonymous use of clinical, instrumental, and photographic data for scientific purposes.

Patients were selected for this study according to the following inclusion criteria:

- Mastectomies performed either prophylactically, after screening for genetic mutations, or following a BC diagnosis,
- Immediate subpectoral breast reconstruction at the time of mastectomy, either with permanent breast implants or with a tissue expander,
- Surgery was to be performed between January 2022 and May 2024.
- The exclusion criteria were as follows:
- Pre-pectoral breast reconstruction,
- Simple mastectomies without immediate reconstruction.

The data for this clinical study were collected retrospectively from the electronic record system of the hospital department, ensuring complete anonymity. Data were extracted from the electronic health record (EHR) system used routinely in the department. This system maintains detailed records of all patient encounters, including clinical evaluations, laboratory and imaging results, medication administration, and operative details. The data collected included the following:

- Preoperative patient characteristics: date of birth, body mass index (BMI), smoking habit, comorbidities such as hypertension, diabetes or anemia, previous breast surgery or radiotherapy, and hemoglobin levels before admission.
- Information about the surgical procedure: therapeutic or prophylactic intent, previous neoadjuvant therapy, surgical technique used (HD or EPS), the type of reconstruction performed. The selection of the type of reconstructive implant was at the discretion of the plastic surgeon, based on the patient’s desire, her clinical characteristics, and the intraoperative clinical evaluation of the viability of the flaps after mastectomy.

Data on the immediate postoperative period, during the inpatient stay in the gynecological department, according to the visual analog scale (VAS) [14]: the assessment of pain on the first, second, and third day of hospitalization; the assessment of drain volume (ml) on the first, second, and third day of hospitalization; the need for hemoglobin transfusions; the hemoglobin value before discharge (for comparison with the value before hospitalization); and the duration of hospitalization. It should be noted that the VAS ranges from a minimum value of 0 to a maximum value of 10, so the pain experienced by the respondent increases as the score increases. Data were collected on patients’ progress in the postoperative period through visits to the plastic surgery outpatient clinic and the gynecology outpatient clinic. Patients were followed for at least thirty days after surgery to detect the onset of any complications. Information collected included the following: the postoperative day of drain removal, the occurrence of early complications (within 30 days) [15], any readmissions determined by the occurrence of complications, and adjuvant therapy. Postoperative complications were graded based on the Clavien–Dindo classification system [16]. Data were collected through reviews of electronic medical records. The Clavien–Dindo classification system for surgical complications divides them into grades based on the severity and treatment required [16]. Grade I complications involved minor deviations from the normal postoperative course. These do not require any pharmacological treatment, surgery, endoscopic, or radiological interventions. Basic therapeutic measures like antiemetics, antipyretics, analgesics, diuretics, electrolytes, and physical therapy are allowed at this level. Wound infections that are treated with bedside procedures also fall under Grade I. Grade II complications require pharmacological intervention with drugs beyond those permitted for Grade I, such as antibiotics. Grade III complications necessitate surgical, endoscopic, or radiological intervention. Grade IV represents life-threatening complications that demand intensive care (ICU) or intermediate care (IC) management. Grade V is the most severe and refers to the patient’s death resulting from complications.

### Statistical Analysis

Statistical analyses were performed using IBM SPSS version 29.0 statistical software. The independent sample *t*-test was used to determine whether there was a statistically significant difference between the means of two groups. For dichotomous variables, the chi-squared test was used. Statistical significance was defined as a *p*-value < 0.05.

## 3. Results

### 3.1. Characteristics of the Population

The analysis included 56 patients who underwent a total of 65 mastectomies, of which 9 were bilateral and 47 were unilateral. Of these mastectomies, 16 were performed for prophylactic reasons, while the remaining 49 were for oncologic indications.

The two different techniques used to dissect were EPS in 39 mastectomies and HD in 26 mastectomies. Table 1 and Table 2 show the characteristics of the study population (one woman and two mastectomies) by the two dissection groups: Group 1 (EPS) and Group 2 (HD).

The two groups were comparable in terms of mean age at surgery, body mass index (BMI), smoking habit, and comorbidities, such as hypertension, anemia, and diabetes. The only difference between the two groups was a greater prophylactic indication in HD patients than in plasma blade patients (*p* = 0.007).

Intrinsic features of the surgery, such as whether the nipple was preserved and the immediate reconstruction with a skin expander or definitive prosthesis, did not differ between the two groups (Table 3).

### 3.2. Postoperative Course

On the day of surgery and for the next two days, the volume of blood and serum in the breast surgical drains was monitored, and patients were asked for a subjective assessment of their pain through the VAS. None of the patients required transfusions, so the postoperative hemoglobin level was compared with that obtained from the hematochemical tests performed during the pre-admission visit, obtaining the hemoglobin drop between the pre-and postoperative values. The total length of hospital stays and the number of days after which the breast drains were permanently removed were collected.

The pain perceived by patients on the first and second postoperative day was significantly greater in patients undergoing surgery with HD than with EPS (*p* = 0.006 and *p* = 0.039, respectively, on day 1 and 2). The duration of hospitalization was also shorter in patients undergoing EPS (*p* = 0.014). (Table 4 women and Table 5 mastectomies).

Early complications were analyzed, considered as all complications diagnosed within 30 days after surgery [15] and in any case before the start of any adjuvant oncological therapy. The complications were found to be consistent with those most frequently discussed in the literature [17].

Neither early complications nor reintervention rates (Clavien–Dindo complication 3) differed by the type of dissection technique (*p* = 0.436 and *p* = 0.548, respectively, Table 6).

## 4. Discussion

This study aims to compare two surgical techniques, Hydrodissection (HD) and electrical plasma surgery (EPS), which are preferred to the more traditional electrosurgical scalpel in the current surgical practice. These two dissection techniques are associated with less trauma to the skin flaps produced during mastectomy, allowing less vascular damage and therefore a greater likelihood of optimal healing.

Studies in the literature comparing electrosurgery with EPS or electrosurgery with HD support the advantages of using these techniques.

EPS is a low-temperature electrosurgical device that generates electrical plasma along its cutting edges using short radiofrequency pulses. Operating at significantly lower temperatures (40–140 °C) than conventional electrosurgical tools, EPS reduces thermal damage to surrounding tissues by 50–90%, achieving precise, scalpel-like cutting with effective hemostasis and minimal collateral impact [7].

Animal and human studies demonstrate several advantages of EPS, including shallower thermal injury, reduced inflammation, stronger wound healing, and improved aesthetic outcomes due to narrower scars [6,18]. The use of EPS appears to be a technique with comparable hemostatic efficacy to traditional electrosurgery, but with enhanced skin flap perfusion [4], less thermal injury depth, reduced inflammatory response, increased wound strength, and reduced scar width associated with a better cosmetic outcome [6,7,18].

HD is a technique first introduced by Worland in 1996 for mastectomy, which uses a blunt cannula to deliver a crystalloid solution to aid in a precise separation of tissue layers [11]. The solution expands the space between Cooper’s ligaments, allowing clearer dissection planes, minimizing tissue injury, and improving flap quality with preserved subcutaneous vascular integrity with fewer re-interventions to improve the cosmetic result when compared to electrosurgery [11]. HD has been shown to decrease perioperative blood loss due to the hydrostatic compression of small vessels [19]. It is considered a safe alternative to standard electrocautery mastectomy in a selected cohort of patients with no additional risk factors for complications [12,20,21].

While research confirms the superiority of both techniques over electrocautery [4,6,7,12,18,21], no direct comparison between them exists. This study addresses this gap by evaluating differences in complications and postoperative outcomes.

The anamnestic parameters analyzed in our study are considered in the literature as risk factors for the occurrence of complications [22] since BMI could influence the thickness of the skin flap [23], and cardiovascular risk factors could influence the vascularization of the flap [22]. In our study, the two groups were similar and comparable for anamnestic characteristics (age, BMI, and comorbidities such as smoking, hypertension, anemia, and diabetes), while HD was more frequently used for prophylactic surgery.

In our center, the type of reconstruction is determined by the plastic surgeon based on the size of both breasts and oncological, radiation, technical, comorbidity, and skin quality factors [24]. In this study, we included only patients undergoing subpectoral breast reconstruction, to avoid having the type of pre-pectoral vs. sub-pectoral breast reconstruction as a confounding factor for complications.

In terms of the postoperative course, pain, as assessed by the VAS, was better in the EPS group on both the first and second day of hospitalization. The pain was extremely low on the first and second day in patients undergoing EPS. These results are consistent with other studies in the literature on EPS, which claim that this technique has excellent results in reducing postoperative wound pain [25]. It was pointed out that, compared to electrosurgery, patients in the EPS group reported a greater reduction in their pain score from the first postoperative day, resulting in no need for analgesic therapy [25].

The difference in the number of hospital days suggests that EPS patients are discharged more quickly. A study conducted by Tasoulis [19] et al. showed a statistically significant difference (*p* = 0.033) between HD and electrosurgery in terms of the length of stay in favor of HD (median 2 [1–3] vs. 2.5 [1–5], respectively). In contrast, in our study, we found a median hospital stay of 5.6 days in the HD group compared to a median of 3.6 days in the EPS group (the day of surgery was also counted).

Differences in the occurrence of complications between the two groups were analyzed through a Clavien–Dindo classification [16]. No Clavien–Dindo 2, 4, and 5 complications were recorded in both groups.

The two techniques were found to overlap in terms of both the overall rate of complications and the rate of surgical re-intervention (Clavien–Dindo 3). In our study, the complication rate of 26% related to the HD technique was slightly higher than that reported in the literature. For instance, in a review of 457 mastectomies with immediate reconstructions performed using HD, the complication rate was 22.5% [26]. Additionally, in a single-institution retrospective evaluation, Abbott et al. reported a 20% complication rate among 113 mastectomies performed with HD [27]. This difference in complication rates could also be explained by a more accurate data collection and recording process in our study. On the other hand, reintervention rates, which are a more objective metric, showed no significant variation from the literature (20% in our study vs. 15.1–23% reported in the literature) [26,27,28].

A strength of this study is that the idea of comparing these two techniques stems from the analysis of validated instruments in the literature such as the VAS and Clavien–Dindo classification and the lack of such a study. The limitations of this study are the small sample size, the lack of randomization of the sample to the two techniques, and the fact that, for HD in the present study, an aqua-dissector was used for the dissection technique instead of a standardized instrument.

## 5. Conclusions

Mastectomy is a widely performed procedure for oncologic or prophylactic indications. Optimizing the surgical technique, using the method with better postoperative functional recovery, shorter hospital stays and lower risks of complications is crucial for the proper management of patients. The results of this study show that demolition mastectomy with EPS is superior to HD in terms of pain in the first two postoperative days and length of the hospital stay. However, this study shows that differences in the immediate postoperative period do not affect the overall rate of complications and the rate of surgical re-intervention.

Further studies comparing these demolition methods with larger sample sizes and randomized study designs are needed to better define the superiority of one technique over the other to ensure that patients receive the best possible approach with the lowest possible risk.

## Figures and Tables

**Table 1:** Characteristics of the study population, women. Group 1 (EPS: electrical plasma surgery) and Group 2 (HD: hydrodissection). BMI: Body mass index; and Hgb: Hemoglobin.

	Group 1 (EPS)	Group 2 (HD)	*p*-Value
	n = 36	n = 20 (%)	
**Mean age at surgery**	52 (S.D. 11)	53 (S.D. 15)	0.667
**BMI (kg/m^2^)**	24.6 (S.D.3.8)	23.9 (S.D. 3.5)	0.5224
**Smokers**	5 (13.9%)	3 (15.0%)	0.909
**Not smokers**	31 (86.1%)	17 (85.0%)	
**Hypertension**	7 (19.4%)	5 (25%)	0.627
**Not hypertension**	29 (80.6%)	15 (75%)	
**Diabetic**	3 (8.3%)	0 (0%)	0.184
**Not diabetic**	33 (91.7%)	20 (100%)	
**Anemic (Hgb < 12 g/dL)**	1 (2.8%)	2 (10%)	0.250
**Non-anemic (Hgb >/= 12 g/dL)**	35 (97.2%)	18 (90%)	

**Table 2:** Characteristics of the study population, mastectomies. Group 1 (EPS: electrical plasma surgery) and Group 2 (HD: hydrodissection).

	Group 1 (EPS)	Group 2 (HD)	*p*-Value
	n = 39 (%)	n = 26 (%)	
**Previous breast surgery**			0.878
- **Yes**	5 (12.8)	3 (11.5)	
- **No**	34 (87.2)	23 (88.5)	
**Previous breast radiation therapy**			0.342
- **Yes**	2 (5.1)	3 (11.5)	
- **No**	37 (94.9)	23 (88.5	
**Indication for surgery**			**0.007**
- **Prophylactic**	5 (13)	11 (42)	
- **Diagnosis of breast cancer**	34 (87)	15 (58)	
**Neoadjuvant chemotherapy**			0.241
- **Yes**	7 (17.9)	2 (7.8)	
- **No**	32 (82.1)	24 (92.2)	

**Table 3:** Characteristics of the surgery. NAC: nipple–areola complex; Group 1 (EPS: electrical plasma surgery) and Group 2 (HD: hydrodissection).

	Group 1 (EPS)	Group 2 (HD)	*p*-Value
	n = 39 (%)	n = 26 (%)	
**Nipple management**			0.244
- **Skin-sparing mastectomy**	16 (41)	7 (26.9)	
- **NAC-sparing mastectomy**	23 (59)	19 (73.1)	
**Immediate reconstruction with skin expander**	33 (85)	17 (65.4)	0.071
**Immediate reconstruction with a permanent prosthesis**	6 (15)	9 (74.5)	

**Table 4:** Immediate postoperative course, women. Group 1 (EPS: electrical plasma surgery) and Group 2 (HD: hydrodissection).

	Group 1 (EPS)	Group 2 (HD)	*p*-Value
	n = 36 (S.D.)	n = 20 (S.D.)	
**Hemoglobin drop between pre- and post-op (g/dL)**	1.6 (1.1)	2.1 (1.1)	0.112
**VAS on the day of surgery (mL)**	2.7 (2.3)	4 (3)	0.083
**VAS on the first day after surgery (mL)**	1.3 (2.2)	3.3 (3)	**0.006**
**VAS on the second day after surgery (mL)**	1.58 (1.9)	3 (2.9)	**0.039**
**Length of hospitalization (days)**	3.6 (0.8)	5.6 (4.7)	**0.014**

**Table 5:** Immediate postoperative course, mastectomies. Group 1 (EPS: electrical plasma surgery) and Group 2 (HD: hydrodissection).

	Group 1 (EPS)	Group 2 (HD)	*p*-Value
	n = 39 (S.D.)	n = 26 (S.D)	
**Volume of surgical drainage on the day of surgery (mL)**	95 (108)	98 (122)	0.939
**Volume of surgical drainage on the first day after surgery (mL)**	231 (98)	203 (122)	0.323
**Volume of surgical drainage on the second day after surgery (mL)**	192 (86)	203 (137)	0.679
**Days between surgery and surgical drain removal**	11.7 (5)	11.6 (3)	0.919

**Table 6:** Early complications and need for re-intervention.

	Group 1 (EPS)	Group 2 (HD)	*p*-Value
	n = 39 (%)	n = 26 (%)	
**Presence of early complications (Clavien–Dindo 1–3)**	10 (26)	9 (34.6)	0.436
**Absence of early complications (Clavien–Dindo 1–3)**	29 (74)	17 (65.4)	
**Surgical reintervention (Clavien–Dindo 3)**	8 (20)	7 (27)	0.548
**No surgical reintervention (Clavien–Dindo 3)**	31 (80)	19 (73)	

## Data Availability

Data are contained within the article.

## References

[1] Sedeta E.T., Jobre B., Avezbakiyev B. (2023). Breast Cancer: Global Patterns of Incidence, Mortality, and Trends. J. Clin. Oncol..

[2] Daly M.B., Pal T., Berry M.P., Buys S.S., Dickson P., Domchek S.M., Elkhanany A., Friedman S., Goggins M., Hutton M.L. (2021). Genetic/Familial High-Risk Assessment: Breast, Ovarian, and Pancreatic, Version 2.2021, NCCN Clinical Practice Guidelines in Oncology. J. Natl. Compr. Cancer Netw..

[3] De Vita R., Buccheri E.M., Villanucci A., Pozzi M. (2019). Breast Reconstruction Actualized in Nipple-Sparing Mastectomy and Direct-to-Implant, Prepectoral Polyurethane Positioning: Early Experience and Preliminary Results. Clin. Breast Cancer.

[4] Habibi M., Prasath V., Dembinski R., Sacks J.M., Rosson G.D., Sebai M.E., Mirkhaef S., Bello R.J., Siotos C., Broderick K.P. (2021). Comparison of Mastectomy and Breast Reconstruction Outcomes Using Low Thermal Dissection versus Traditional Electrocautery: A Blinded Randomized Trial. Breast Cancer Res. Treat..

[5] Gabriel A., Maxwell G.P. (2017). Prepectoral Breast Reconstruction in Challenging Patients. Plast. Reconstr. Surg..

[6] Ruidiaz M.E., Messmer D., Atmodjo D.Y., Vose J.G., Huang E.J., Kummel A.C., Rosenberg H.L., Gurtner G.C. (2011). Comparative Healing of Human Cutaneous Surgical Incisions Created by the PEAK PlasmaBlade, Conventional Electrosurgery, and a Standard Scalpel. Plast. Reconstr. Surg..

[7] Loh S.A., Carlson G.A., Chang E.I., Huang E., Palanker D., Gurtner G.C. (2009). Comparative Healing of Surgical Incisions Created by the PEAK PlasmaBlade, Conventional Electrosurgery, and a Scalpel. Plast. Reconstr. Surg..

[8] Rusby J.E., Smith B.L., Gui G.P.H. (2010). Nipple-sparing mastectomy. Br. J. Surg..

[9] Vore S.J., Wooden W.A., Bradfield J.F., Aycock E.D., Vore P.L., Lalikos J.F., Hudson S.S. (2002). Comparative healing of surgical incisions created by a standard “Bovie”, the Utah Medical Epitome Electrode, and a Bard-Parker cold scalpel blade in a porcine model: A pilot study. Ann. Plast. Surg..

[10] Bille C., Dalaei F., Thomsen J.B. (2019). Identifying the dissection plane for mastectomy—Description and visualization of our technique. Gland. Surg..

[11] Worland R.G. (1996). Expanded Utilization of the Tumescent Technique for Mastectomy. Plast. Reconstr. Surg..

[12] Khavanin N., Fine N.A., Bethke K.P., Mlodinow A.S., Khan S.A., Jeruss J.S., Hansen N.M., Kim J.Y.S. (2014). Tumescent technique does not increase the risk of complication following mastectomy with immediate reconstruction. Ann. Surg. Oncol..

[13] Sowa Y., Inafuku N., Kodama T., Morita D., Numajiri T. (2018). Preventive effect on seroma of use of PEAK PlasmaBlade after latissimus dorsi breast reconstruction. Plast. Reconstr. Surg. Glob. Open.

[14] Heller G.Z., Manuguerra M., Chow R. (2016). How to Analyze the Visual Analogue Scale: Myths, Truths and Clinical Relevance. Scand. J. Pain.

[15] Metere A., Fabiani E., Lonardo M.T., Giannotti D., Pace D., Giacomelli L. (2020). Nipple-Sparing Mastectomy Long-Term Outcomes: Early and Late Complications. Medicina.

[16] Dindo D., Demartines N., Clavien P.A. (2004). Classification of Surgical Complications: A New Proposal With Evaluation in a Cohort of 6336 Patients and Results of a Survey. Ann. Surg..

[17] Masià J., iBAG Working Group (2020). The Largest Multicentre Data Collection on Prepectoral Breast Reconstruction: The iBAG Study. J. Surg. Oncol..

[18] Chang E.I., Carlson G.A., Vose J.G., Huang E.J., Yang G.P. (2011). Comparative Healing of Rat Fascia Following Incision with Three Surgical Instruments. J. Surg. Res..

[19] Tasoulis M.K., Agusti A., Karakatsanis A., Montgomery C., Marshall C., Gui G. (2019). The Use of Hydrodissection in Nipple- and Skin-Sparing Mastectomy: A Retrospective Cohort Study. Plast. Reconstr. Surg. Glob. Open.

[20] Rica M.A.I., Norlia A., Rohaizak M., Naqiyah I. (2007). Preemptive Ropivacaine Local Anaesthetic Infiltration Versus Postoperative Ropivacaine Wound Infiltration in Mastectomy: Postoperative Pain and Drain Outputs. Asian J. Surg..

[21] Shoher A., Hekier R., Lucci A. (2003). Mastectomy Performed with Scissors Following Tumescent Solution Injection. J. Surg. Oncol..

[22] Paprottka F.J., Schlett C.L., Luketina R., Paprottka K., Klimas D., Radtke C., Hebebrand D. (2019). Risk Factors for Complications after Skin-Sparing and Nipple-Sparing Mastectomy. Breast Care.

[23] Robertson S., Jeevaratnam J., Agrawal A., Cutress R. (2017). Mastectomy Skin Flap Necrosis: Challenges and Solutions. Breast Cancer Targets Ther..

[24] Gilmour A., Cutress R., Gandhi A., Harcourt D., Little K., Mansell J., Murphy J., Pennery E., Tillett R., Vidya R. (2021). Oncoplastic Breast Surgery: A Guide to Good Practice. Eur. J. Surg. Oncol..

[25] Huang C.Y., Chiu H.Y., Lai H.W., Chen D.R. (2016). The PEAK Plasma Blade May Reduce Postoperative Analgesic Medication Use in Breast Cancer Surgery. Breast Cancer Surg..

[26] Seth A.K., Hirsch E.M., Fine N.A., Dumanian G.A., Mustoe T.A., Galiano R.D., Hansen N.M., Kim J.Y.S. (2011). Additive Risk of Tumescent Technique in Patients Undergoing Mastectomy with Immediate Reconstruction. Ann. Surg. Oncol..

[27] Abbott A.M., Miller B.T., Tuttle T.M. (2012). Outcomes after Tumescence Technique versus Electrocautery Mastectomy. Ann. Surg. Oncol..

[28] Vargas C.R., Koolen P.G., Ho O.A., Ricci J.A., Tobias A.M., Lin S.J., Lee B.T. (2015). Tumescent Mastectomy Technique in Autologous Breast Reconstruction. J. Surg. Res..

